# Metabolic Profile Discriminates and Predicts *Arabidopsis* Susceptibility to Virus under Field Conditions

**DOI:** 10.3390/metabo11040230

**Published:** 2021-04-09

**Authors:** Bernadette Rubio, Olivier Fernandez, Patrick Cosson, Thierry Berton, Mélodie Caballero, Roxane Lion, Fabrice Roux, Joy Bergelson, Yves Gibon, Valérie Schurdi-Levraud

**Affiliations:** 1 Université de Bordeaux, INRAE, Biologie du Fruit et Pathologie, UMR 1332, F-33140 Villenave d’Ornon, France; bernadette.rubio@inrae.fr (B.R.); olivier.fernandez@univ-reims.fr (O.F.); patrick.cosson@inrae.fr (P.C.); thierry.berton@laposte.net (T.B.); melodie.caballero@inrae.fr (M.C.); roxane.lion@inrae.fr (R.L.); yves.gibon@inrae.fr (Y.G.); 2 CNRS, INRAE, Université de Toulouse, LIPM, F-31320 Castanet-Tolosan, France; fabrice.roux@inrae.fr; 3 Ecology & Evolution, University of Chicago, 1101 E 57th St, Chicago, IL 60637, USA; jbergels@uchicago.edu

**Keywords:** *Arabidopsis thaliana*, field conditions, growth, central metabolism, specialized metabolism, trade-off, *Turnip mosaic virus*

## Abstract

As obligatory parasites, plant viruses alter host cellular metabolism. There is a lack of information on the variability of virus-induced metabolic responses among genetically diverse plants in a natural context with daily changing conditions. To decipher the metabolic landscape of plant-virus interactions in a natural setting, twenty-six and ten accessions of *Arabidopsis thaliana* were inoculated with *Turnip mosaic virus* (TuMV), in two field experiments over 2 years. The accessions were measured for viral accumulation, above-ground biomass, targeted and untargeted metabolic profiles. The phenotypes of the accessions ranged from susceptibility to resistance. Susceptible and resistant accessions were shown to have different metabolic routes after inoculation. Susceptible genotypes accumulate primary and secondary metabolites upon infection, at the cost of hindered growth. Twenty-one metabolic signatures significantly accumulated in resistant accessions whereas they maintained their growth as mock-inoculated plants without biomass penalty. Metabolic content was demonstrated to discriminate and be highly predictive of the susceptibility of inoculated *Arabidopsis*. This study is the first to describe the metabolic landscape of plant-virus interactions in a natural setting and its predictive link to susceptibility. It provides new insights on plant-virus interactions. In this undomesticated species and in ecologically realistic conditions, growth and resistance are in a permanent conversation.

## 1. Introduction

Plant health is of primary importance to improve and secure food supply for an increasing human population. Plant viruses represent the major taxonomic group of emergent pathogens of plants [1], and viral infection is one of the most alarming biotic threats due to the impact of climate change on the spatial and temporal distribution of vectors and viruses [2,3]. Compared to other plant pathogens, viruses are particularly unpredictable and difficult to combat. In this context, an understanding of the response of plants to viral infection has great importance in sustainable agricultural solutions.

The genus *Potyvirus*, to which the turnip mosaic virus (TuMV) belongs, is one of the largest genera among plant viruses, causing considerable economic damage in vegetable and fruit crops worldwide [4]. The completion of the viral multiplication and movement cycle results from a complex interplay between virus- and host-encoded factors that can have profound impacts on plant fitness. To invade plants, those obligatory parasites have developed tactics to reroute host cellular functions and components for their own benefits. Whatever the outcome of the interaction—compatibility, leading to disease, or incompatibility, leading to resistance—massive reprogramming of metabolism is observed. Indeed, as viruses perturb and exploit the host’s carbon [5] and nitrogen [6] metabolism to make their own compounds, demand for energy within the plant increases to sustain viral multiplication, systemic spread and defense responses [7].

To date, viral impacts on plant metabolism have been mainly studied in experiments conducted in controlled conditions and on a limited diversity of host genotypes. In these conditions, respiration [8], photosynthetic efficiency [9] and carbon partitioning [6] were shown to be modified. For example, in a susceptible interaction between tobacco and potato virus Y (PVY), both an increase of soluble carbohydrates and a decrease of photosynthesis were observed 4 days after inoculation (dai) [10]. These observations were confirmed by the demonstration that potato leaves exhibit a decrease in sugar levels one day after PVY infection, with a subsequent increase in both inoculated and systemic leaves a few days later ([5,11]). Similarly, inoculation of susceptible hosts with cucumber mosaic virus (CMV) results in a localized reduction in starch accumulation as a consequence of altered carbohydrate metabolism at viral infection sites [12], although starch accumulates to high levels in systemically infected leaves [10]. Increases in amino acid content have also been demonstrated in terms of total or individual amino acids, as well as in polyamines, in a variety of systems (reviewed in [6,13,14]). Viruses interfere with fatty acids, structural components of intracellular membranes in which replication can take place [15]. They also exploit transport systems in order to invade cells in systemic tissues away from the initial site of infection. This is achieved through an interaction between viral proteins and components of the long distance transport machinery like phloem proteins [16]. Like primary metabolism, specialized metabolism is strongly impacted due to its participation in multiple defense signaling cascades [17]. Numerous compounds, including hormones, are involved in defense ([18,19]). Among them, glucosinolates and phytoalexins play significant roles in defense against a range of pathogens [20].

In a compatible interaction, the outcome of viral colonization can include symptoms such as stunting, chlorosis or necrosis depending on the pathosystem. Incompatible interactions trigger plant resistance and defense signaling that involve the action of antimicrobial components and specific defense proteins [17]. These defense responses are now widely acknowledged to involve a trade-off in model plants, with the cost of resistance generating a negative impact on plant fitness [21,22,23,24,25,26,27]. Similarly, in crop plants, high levels of resistance are often associated with yield penalties [28,29].

While extremely informative, these studies performed under controlled optimal conditions reduce environmental effects and increase the likelihood of finding strong relationships between metabolite levels variations [30]. Thereby, it obscures the complexity and the variability of metabolic responses among genetically diverse plants in a natural context where they have to face multiple stresses. Even in main crop species, large-scale metabolic profiling of field-grown populations of genetically diverse accessions remains rare [31,32,33].

In *Arabidopsis thaliana*, metabolite profiling of large populations of natural accessions or inbred lines has allowed the identification of descriptor sets of metabolites that are predictive of biomass [32,34,35] and physiological traits such as freezing tolerance [36] and herbivore resistance [37]. But, none of these studies addressed the growth and metabolic response to biotic stress in a changing environment.

Here, following the modern standards of ecological genomics [38], we aimed at improving our understanding of the virus-induced reprogramming of metabolism through deciphering variation in the metabolic landscape of the natural *Arabidopsis thaliana*/turnip mosaic virus (TuMV) pathosystem in growth conditions close to environmental reality. To do so, we set up two experiments in the field and explored targeted and untargeted metabolic profiles on 26 accessions and 10 accessions, in 2015 and 2017 respectively. These accessions spanned responses ranging from high susceptibility to full resistance. This work aimed to (i) decipher the trade-offs among responses to TuMV, metabolism and growth in field conditions in contrasted *Arabidopsis* accessions, (ii) characterize the metabolic disturbances and its kinetics for a contrasted set of accessions, and (iii) identify discriminant metabolic biomarkers of susceptibility/resistance to viruses in *A. thaliana*.

## 2. Results

### 2.1. In the Field, Susceptible Accessions Accumulate PRIMARY METABolites upon Infection, at the Cost of Hindered Growth, Whereas Resistant Accessions Grow with Limited Changes

To analyze the relationship between infection, growth and central metabolism, a set of 26 accessions [39] were mock and TuMV-inoculated in a field experiment in 2015. Thirteen days after inoculation (dai), the viral load of the samples, their above-ground dry biomass and their composition for 10 metabolites features were evaluated.

The quantification of the viral load by ELISA confirmed that eight genotypes out of twenty-six TuMV-inoculated were considered fully resistant as no virus was detected (Table 1). The remaining eighteen accessions fell within a range of susceptibility, OD from 0.258 to 0.66 (Table 1).

**Figure 1:**
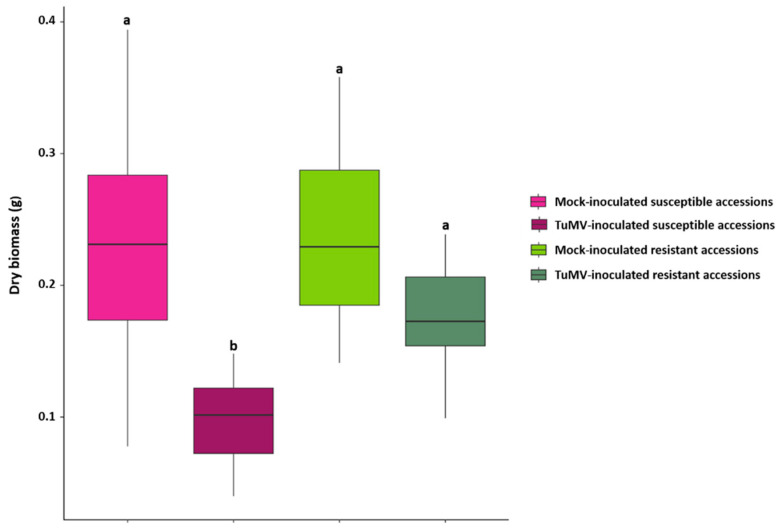
Above ground Dry biomass for mock-inoculated and TuMV-inoculated susceptible and resistant accessions measured at 13 days after inoculation (dai) on 26 accessions in the ‘2015′ field experiment. Statistical comparisons (Kruskal-Wallis test) on dry biomass were performed between all categories of accessions (N = 21 for Mock and inoculated susceptible categories; N = 8 for mock and inoculated resistant categories). Plots with the same letter are not significantly different at *p* = 0.05.

Aboveground dry biomass was measured on mock-inoculated and TuMV-inoculated plants. Aboveground dry biomass was significantly lower for the 18 inoculated susceptible accessions compared to those susceptible accessions when treated only with a mock solution, indicating that infection led to a decrease in the growth of susceptible plants. There was no significant difference between the aboveground dry biomass of the eight resistant inoculated accessions compared to their mock-inoculated counterparts (Kruskal-Wallis test; Figure 1).

Ten key metabolic features, amino acids, proteins, glutamate, malate, fumarate, starch, glucose, fructose, sucrose and chlorophyll a, were measured in 2015 for each accession. Thirteen days after inoculation, analysis of the Spearman correlation pattern between dry biomass, OD and the content in the different metabolites features (Table S1 in Supplementary Materials) showed that that viral accumulation was negatively correlated with dry aboveground biomass. In mock-inoculated samples, a significant positive correlation was found between malate, fumarate, starch, glucose, fructose, sucrose and aboveground dry biomass whereas a negative significant correlation was found with amino acids, proteins, glutamate (Table S1). Compared to mock-inoculated plants, the remodelling of metabolic contents in TuMV-inoculated plants was shown as biomass appeared negatively correlated with nine out of 10 of the measured metabolites features (all except chlorophyll a). Whereas, on the contrary, the viral load appears positively correlated to these same nine metabolites’ (amino acids, proteins, glutamate, malate, fumarate, starch, glucose, fructose and sucrose) features (Table S1).

**Figure 2:**
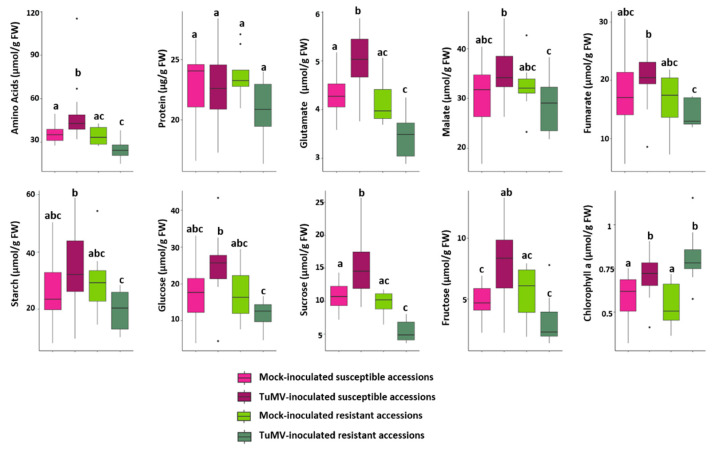
Comparisons of central metabolic content in mock-inoculated and TuMV-inoculated resistant and susceptible *A. thaliana* accessions in the 2015 field experiment. Boxplot sharing the same letter are not significantly different, at the chosen level (default 5%).

A detailed analysis showed that the 18 TuMV-inoculated susceptible accessions exhibited a significant accumulation of eight out of 10 central metabolites (i.e., amino acids, glutamate, malate, fumarate, starch, glucose, fructose and sucrose), when compared to either the eight resistant inoculated accessions or the set of mock inoculated controls (Figure 2). The chlorophyll content in TuMV-inoculated susceptible and resistant accessions was not significantly reduced, confirming that sampling occurred prior to macroscopic symptoms of chlorosis.

TuMV inoculated susceptible accessions are represented by pink boxplot (light pink for Mock-inoculated and dark pink for TuMV inoculated) and resistant accessions by green boxplots (light green for Mock-inoculated and dark green for TuMV inoculated). Statistical analyzes were performed on each of the 10 metabolites features (according to the normality of the data, ANOVA or Kruskall-Wallis test was performed). For each metabolite, plots with the same letter are not significantly different at *p* = 0.05.

The TuMV-inoculated susceptible genotypes therefore accumulate the majority of the measured metabolites features without being used for their growth, whereas, in addition to maintaining their growth, the inoculated resistant accessions exhibited similar amounts of metabolites features relative to those observed for mock-inoculated controls.

### 2.2. Metabolic Content Discriminates Inoculated A. thaliana Susceptible and Resistant Accessions

**Figure 3:**
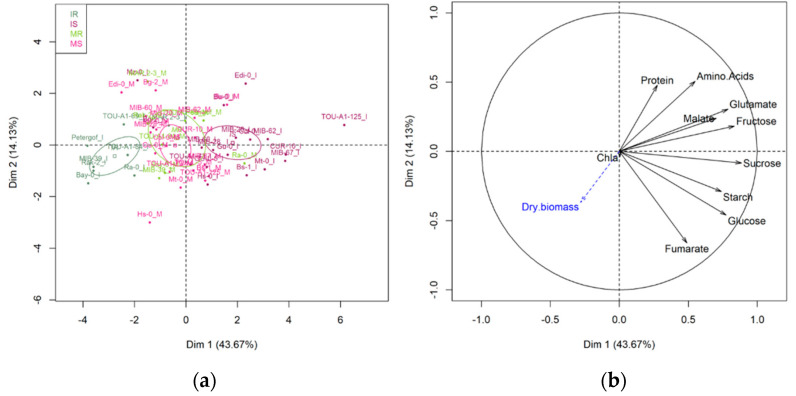
Principal component analysis (PCA) performed on 26 *A. thaliana* accessions with 10 primary metabolic traits measured at 13 dai on mock-inoculated plants and TuMV-inoculated plants in the ‘2015’ field experiment. (**a**) Score plot of individuals. Score plots of resistant accessions are in green, light green for mock-inoculated, dark green for TuMV-inoculated. Score plots of susceptible accessions are in pink, light pink for mock-inoculated, dark pink for TuMV-inoculated. The confidence ellipses around the centroid of individuals are represented. (**b**) Variable correlation graph. Dry biomass was considered as explanatory traits (in blue on the variable factor map). Chla = Chlorophyll a.

Principal component analysis (PCA) performed with the 10 primary metabolic traits measured at 13 days after inoculation on mock-inoculated and TuMV-inoculated accessions showed that targeted metabolism does not distinguish between susceptible and resistant genotypes when mock- inoculated (Figure 3a).

While metabolic change lead to a clear differentiation between susceptible and resistant accessions that have been inoculated with TuMV (Figure 3a), the first axe of the PCA discriminating between susceptible and resistant accessions (46.67% of the explained variance).

The contribution of primary metabolites also differed between mock-inoculated plants and TuMV-inoculated plants with clear contribution of most of the primary metabolites to response in susceptible genotypes (Figure 3b).

### 2.3. Metabolic Differentiation between Mock-Inoculated and Tumv-Inoculated Genotypes Appears as Early as 5 Days after Inoculation

In order to specify the kinetics of setting up the metabolic differentiation between susceptible and resistant genotypes, TuMV-inoculated and mock, a field experiment was conducted in 2017. Five resistant genotypes and five susceptible genotypes from the 2015 set were mock-inoculated or inoculated with TuMV. The plants were collected on the day of inoculation (T0) and at 5, 7, 9 and 13 days after inoculation. The viral load was measured at each sampling time. The optical density data taken at 13 dai (Table S2) confirm the classification between susceptible and resistant observed in 2015 for these genotypes.

**Figure 4:**
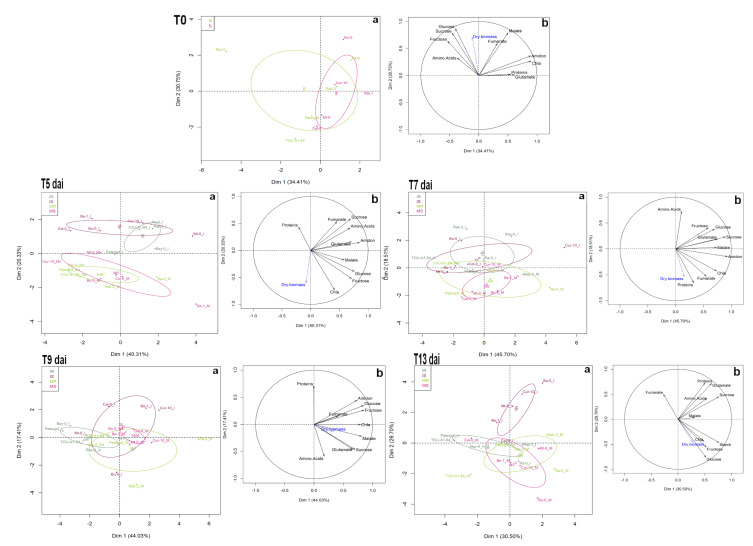
Principal component analysis (PCA) performed with 10 primary metabolic traits measured at 0 (T0), 5 (T5), 7 (T7), 9 (T9) and 13 (T13) days after inoculation on 10 *A. thaliana* accessions mock and TuMV inoculated in the ‘2017’ field experiment. **a** Score plot of individuals. Score plots of resistant accessions are in green, light green for mock-inoculated, dark green for TuMV-inoculated. Score plots of susceptible accessions are in pink, light pink for mock-inoculated, dark pink for TuMV-inoculated. The confidence ellipses around the centroid of individuals are represented. **b** Variable correlation graph. Dry biomass was considered as explanatory traits (in blue on the variable factor map). Chla = Chlorophyll a.

The PCA carried out using the measurement data of the 10 central metabolites show that at T0, in a non-inoculated situation, the metabolic composition does not allow to distinguish between susceptible and resistant genotypes (Figure 4 T0a). Sugars, fumarate and malate appear positively correlated and clearly participate in the biomass (Figure 4 T0b).

Five days after inoculation, a clear differentiation appears between TuMV-inoculated genotypes and mock-inoculated genotypes, independently of the resistance or susceptibility status (Figure 4 T5a). At 7 and 9 days after inoculation, the pattern appears less clear but the mock-inoculated genotypes, resistant or susceptible, still remain grouped (Figure 4 T7 and T9a) whereas susceptible and resistant genotypes TuMV-inoculated have a different metabolic time course. Thirteen days after inoculation, and as already observed in 2015, the clear differentiation, in terms of metabolic composition, between inoculated resistant genotypes and inoculated susceptible genotypes is in place (Figure 4 T13a).

### 2.4. The Identification of Metabolic Predictors Reveals 21 Metabolic Signatures Significantly Accumulated in Resistant Accessions

To analyze the pattern of infection and its relationship to a larger set of metabolites, including specialized metabolites, we conducted an untargeted metabolic analysis by UHPLC-LTQ Orbitrap on the set of 26 accessions showing a contrasting response to TuMV in the 2015 field experiment. This analysis captured a total of 505 workable metabolic signatures (*m*/*z*), corresponding primarily to non-polar specialized metabolites.

As previously observed with targeted central metabolism, there was no difference between susceptible and resistant accessions when mock-inoculated (Figure S1a). In contrast, when inoculated with TuMV, principal component analysis on the metabolic variables clearly separated two groups of accessions according to their susceptibility to the TuMV (Figure S1b).

An orthogonal partial least squares-discriminant analysis (OPLS-DA) was then performed to maximize the variation between the two groups of accessions, mock and TuMV-inoculated, and determine the most significant variables contributing to this variation, i.e., variable importance in the projection (VIPs). OPLS-DA analysis was carried out using the central and specialized metabolite data. The quality of the model was validated by the Q^2^ parameter (goodness-of-prediction parameter) with a value of 0.846, thereby showing high predictive capabilities (Figure S2).

The OPLS-DA analysis performed between the mock- and TuMV-inoculated samples revealed 63 common discriminant metabolic variables (Table S3), most of which (58/63) accumulated in TuMV-inoculated samples and particularly in susceptible accessions (54/58; Table S3). It is worth noting that four VIPs (433;700, 361;491, 512;491 and 64;395) are significantly found in resistant accessions.

To examine which metabolic variables strongly contribute to the OPLS-DA model in TuMV-inoculated susceptible and resistant accessions, variables were ranked according to their VIP values (Table 2).

This ranking confirmed the major role of some metabolites features, including sucrose, glucose, fructose, glutamate, amino acids and fumarate, in the response of susceptible accessions. Of the 140 VIP values identified by OPLS-DA, 119 accumulated to higher levels in susceptible accessions and 21, among which the four VIPs (VIP 433;700, VIP 361;491, VIP 512;491 and VIP 64;395) are found, accumulated to higher levels in resistant accessions (Table 2). The fold change susceptible/resistant reached up to 45 times whereas the fold change resistant/susceptible reached up to 244 times. It is very interesting to highlight two VIPs, VIP 324;184 and VIP 433;482 that are significantly accumulated in resistant accessions when inoculated with a fold-change of 106.8 and 244, respectively, compared to the susceptible accessions.

**Figure 5:**
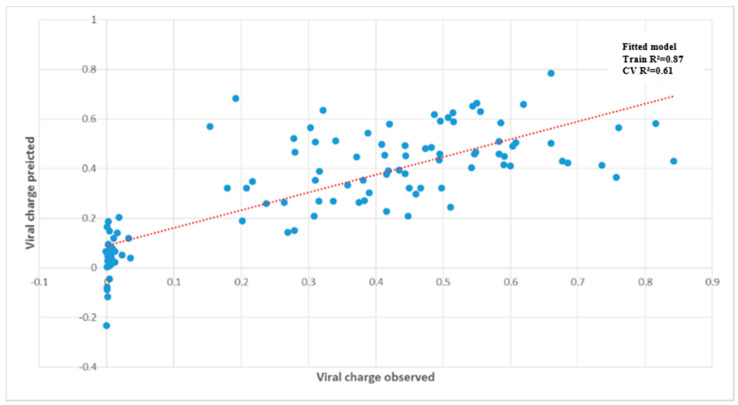
Prediction of viral accumulation by the metabolite matrix on 26 *A. thaliana* accessions in the ‘2015′ field experiment.

A complementary analysis, Partial Least Square (PLS), on the dataset obtained with the 26 accessions was performed to test whether viral accumulation can be predicted from metabolic data. Analysis was performed on the optical density values corresponding to the viral accumulation measured by DAS ELISA for each accession (Figure 5). The PLS coefficient estimated in the training data set revealed, after cross-validation, a correlation of 0.61 between predicted and true viral accumulation, confirming the high predictive power of metabolic composition for TuMV susceptibility.

The metabolite matrix is composed by 10 primary metabolic traits and 505 metabolic signatures (*m/z*). The replicates of the 26 *A. thaliana* accessions are represented by the blue dots (total data point = 98). The dashed linear red line represents the exact prediction.

Among the best 50 VIP-PLS values, 44 were found common to the VIP values obtained with the OPLS-DA analysis (Table S4). In both analyses, the central metabolites sucrose, glucose and glutamate were found to discriminate susceptible and resistant accessions, ranking among the best VIP values (Table S4). Six common VIPs (VIP 394;108, VIP 351;184, VIP 324;184, VIP 280;184, VIP 432;184 and VIP 86;184) detected by the two analyses are found accumulated more significantly in resistant accessions.

## 3. Discussion

In this study, we characterized the metabolic response of *A. thaliana* to its natural viral pathogen, turnip mosaic virus (TuMV). This study is unusual in studying the metabolic response of a variety of accessions to an important naturally occurring virus on *A. thaliana* [42] and its close relative, *A. halleri* [43], in natural settings.

In neither year of the study, from time 0 to 13 days after inoculation, could we distinguish the metabolic profile of TuMV-susceptible and TuMV-resistant accessions grown in the absence of TuMV. This suggests that there are no constitutive metabolic patterns associated with resistance or susceptibility. Nevertheless, these mock-inoculated samples provided an opportunity to describe elements of central metabolism in *A. thaliana* under common garden field conditions. We found that dry biomass was significantly correlated with fumarate concentrations. Fumarate can accumulate to high levels in *A. thaliana* relative to other plant species, suggesting that it likely constitutes a significant fraction of the fixed carbon in *A. thaliana* rosette leaves [44]. Indeed, the amount of carbon stored in fumarate is similar to that accumulated in starch [44]. This is perhaps not surprising because fast-growing plant species such as *A. thaliana* contain significantly higher concentrations of organic acids, such as fumarate, compared to slow-growing plants [44,45], especially under high light intensity conditions such as in our field experiments [45]. We also found that dry biomass was correlated to glucose. Both fumarate and glucose reached high levels in fast growing accessions, 23 and 19 mM, respectively, suggesting that they might be involved in turgor dynamics and thus growth by cellular expansion [46]. In contrast, we detected a negative correlation between dry biomass and protein. Although speculative, this might follow from the fact that lower protein synthesis contributes to increased efficiency of carbon use, because protein synthesis is a costly process [47,48]. This negative relationship is in agreement with previous observations among a collection of *A. thaliana* accessions grown under greenhouse/growth chamber and in controlled conditions [49].

Upon viral infection, in the field, the effect of TuMV inoculation could be observed as early as 5 days after inoculation. At 13 dai, susceptible accessions were clearly distinguished from the resistant ones by the accumulation of central metabolites. Eight out of ten metabolic features (amino acids, glutamate, malate, fumarate, starch, glucose, fructose and sucrose), were strongly associated with viral accumulation. Previous efforts to describe the massive reprogramming of the plant central metabolism that takes place in response to pathogens has focused on fungi and bacteria [50]. Here, we add to the limited literature on plant responses to viral infection, which is restricted to controlled laboratory conditions. For example, in a study of the central metabolic response of *Arabidopsis* to tobacco rattle virus (TRV), Fernandez-Calvino and collaborators [6] showed that the susceptible Col-0 accession significantly accumulated sucrose at 8 days’ post inoculation. Amino acids were also globally accumulated in infected plants compared to mock plants whereas neither starch nor fumarate were accumulated in infected plants. Differences in the virus species, the number of accessions observed and the fact that theirs was a laboratory experiment could explain these different results. Second, in a controlled multi-stress experiment (including TuMV, drought and heat), accumulation of soluble sugars was again observed in Col-0 plants [51]. The strong metabolic disturbances generated in susceptible plants by TuMV infection suggests that the viral infection stimulated the accumulation of major central metabolites, probably as a result of an imbalance between photosynthesis and growth, which is known to favor oxidative stress [52]. Susceptible accessions were also found to accumulate a large number of specialized compounds. In addition to the fact that such an accumulation was apparently ineffective, the synthesis of specialized compounds is expensive in energy and, even if somewhat controlled [53], accumulation could have toxic effects.

Under the field conditions used in our experiments, inoculated resistant accessions were able to grow at the same rate as controls. As biomass is considered as appropriate proxy for fitness under many circumstances [54], it seems to indicate that resistance was achieved with no penalties on measured traits. Unlike susceptible accessions, resistant accessions accumulate a limited number of specialized metabolites in response to the virus, but in much higher quantities than can be observed in susceptible accessions. This contrasts with a study conducted under controlled conditions on the metabolic response of tomato to tomato yellow leaf curl virus (TYLCV) which found that the defense response of resistant lines is effective through the accumulation of many specialized metabolites [55]. The high predictive power of metabolic composition for TuMV susceptibility when infected might enable this composition to serve as a biomarker [30], as has been done for resistance to *Fusarium graminearum* in wheat [56] and for susceptibility to esca disease in grape [57].

The complex molecular network underlying the balance between growth and immunity has been described [24,25,58] mainly in terms of opposition. Recent reviews [26,59] have emphasized, however, that in a natural context, growth and immunity are in a constant conversation. Our study supports this alternative model and highlights new results on *Arabidopsis*/virus interaction. In particular, we have highlighted the very different metabolic paths between susceptible and resistant genotypes. We found that susceptible accessions experience a large accumulation of central and specialized metabolites with a reduction of growth whereas resistant accessions appear capable of continued growth with a targeted metabolic response. Some compounds as VIP 324;184 and VIP 433;482, that presented high fold-change in resistant accessions compared to susceptible ones are of particular interest. Preliminary putative annotation of major metabolic markers was performed using RT, accurate *m*/*z* detected by high-resolution MS and MS2 fragments as described previously [60,61]. The resulting predicted molecular formula were screened through chemical databases (HMDB, METLIN, MassBank) to match putative metabolite identification. The first results show that some of them are phenolic compounds including flavonoids and coumarins. The anti-viral properties of some of these compounds such as quercetagetin or other flavonoids have been demonstrated against Tomato bushy stunt virus [62] and Tobacco mosaic virus [63].

It would be of great interest completing and refining the characterization of these specialized compounds and their biosynthetic pathways, and then to test their involvement in resistance by using pharmacological and/or genetic approaches. It is worth mentioning that anti-phage specialized metabolites molecules, able to block phage replication, have recently been found in *Streptomyces* [64]. This would allow for better understanding the complexity of the underlying mechanisms involved in plants’ responses to viruses in the field and to propose new ideotypes.

## 4. Materials and Methods

### 4.1. Plant Material

Genotypes from a worldwide collection of natural accessions of *Arabidopsis thaliana* were used in our experiments [40]. Twenty-six accessions were challenged with TuMV during the first field experiment in 2015 and then a subset of 10 accessions were selected for a second field experiment in 2017. This subset was selected based on their OD value following TuMV inoculation (listed in Table 1 and Table S2) to represent extreme phenotypes from highly susceptible (S) to resistant (R). To prevent misidentifying accessions as resistant due to inefficient mechanical inoculation, only accessions presenting a resistance phenotype in all our common garden experiments (see [39]) were selected as resistant accessions.

### 4.2. Virus Material

Turnip mosaic virus isolate UK1 [65] was routinely propagated by mechanical inoculations on turnip plants *Brassica rapa* L. ssp *rapa* NA FR 490,001 provided by the BraCySol germplasm center (Ploudaniel, France). To prepare the inoculum, three-week old turnip plants were mechanically inoculated. Symptoms appeared two weeks later. Young symptomatic leaves of five-week-old turnip were then collected to produce the inoculum.

### 4.3. Experimental Design and Growth Conditions

Two common garden experiments were conducted in 2015 (N = 26 *A. thaliana* accessions) and 2017 (N = 10). Experiments realized in 2015 were organized in a randomized complete block design (RCBD). The experiment performed in 2015 contained four blocks with four replicates per accession per block. In 2017, a kinetic analysis was carried out. Thus, five sampling were performed. The first sampling was done the day of inoculation (named thereafter 0 days after inoculation (dai)), and at 5, 7, 9 and 13 (dai). The accessions were arranged in a randomly complete block design of three blocks with three replicates per accession per sampling date.

As described in [39], seedling trays of 40 wells were used. Seeds were sown on 23 March 2015 and on 20 March 2017 in professional horticultural soil (106Scope, PletrAcom, Arles, France) under a cold-frame glasshouse without additional light or heating to ensure homogeneity of germination. At three weeks of age, plants were acclimatized under an opened tunnel before their transfer to the common garden. Plants were inoculated during the acclimation step just before their transfer. Soil of the common garden had been tilled so that seedling trays could be slightly buried. Because the bottoms of the wells were pierced, roots were able to reach the soil. Climate data were recorded over the duration of each experiment (Figure S3a,b). The analysis of climatic data across the three growing environments (cold frame greenhouse, then tunnel, then field) revealed no significant difference between 2015 and 2017 in the two first steps (cold frame greenhouse and tunnel) but a significant difference between 2015 and 2017 for temperatures, rainfall and PAR (photosynthetically active radiation) in common garden (Figure S3c–e).

### 4.4. TuMV Inoculation Procedure and Harvest

One gram of fresh turnip leaves was ground in three volumes of disodium phosphate (Na_2_HPO_4·_12H_2_O) 30 mM and 0.2% diethyldithiocarbamic acid (DIECA). The inoculum was clarified through 10 min centrifugation at 13,000× *g*. Supernatant was recovered and maintained at 4 °C until *Arabidopsis*’ inoculation, which was performed when plants were 4 weeks old and at a 8–10 leaf stage, corresponding to 1.09, 1.10 Boyes stage [66]. Four young expanded leaves of each plant were mechanically inoculated with 20 µL of inoculum with carborundum added on each leaf. Ten minutes after inoculation, plants were rinsed with water. Mock treatments, in which plants were treated exactly as inoculated plants except for the absence of the virus, were also included. The viral concentration of the TuMV inoculum was quantified after inoculation by quantitative PCR [39]. The viral concentrations of the inocula used was 0.049 g/µL in 2015 and 0.269 ng/µL in 2017 common garden experiments (significantly different through a Wilcoxon-Mann Whitney test *p* = 0.029).

According to previous experiments [39], samples were collected 13 days after inoculation (dpi) in the 2015 experiment at the very beginning of the onset of the first symptoms. In 2017, the first sampling was done the day of inoculation (named thereafter 0 days after inoculation (dai)), and at 5, 7, 9 and 13 (dai). The harvest was done in the morning, at the same time for each year of experiment. All rosette leaves above the inoculated leaves (systemic leaves) were collected in vials from Zinsser Analytic^®^ (Eschborn, Germany). Samples were deep-frozen, ground and stored at −80 °C.

### 4.5. Quantification of Viral Accumulation

Viral accumulation was estimated for each individual plant using 100 mg of *A. thaliana* powder in a semi-quantitative double antibody sandwich assay (DAS-ELISA) with a commercial anti-potyvirus monoclonal antibody kit (Agdia-Biofords, Evry, France). The reaction of the substrate (*p*-nitrophenyl phosphate) was followed at 405 nm. Optical densities (OD) were calculated by removing the mean OD value from the healthy *A. thaliana* Col-0 control and normalized using a Col-0 positive control deposited on each ELISA plate. As determined from the ODs of the *A. thaliana* Col-0 positive controls, and in order to avoid overflow, the 15 min measurements were retained. For each accession, we classified its degree of resistance or susceptibility based on the average of the ODs obtained across replicates (Table 1; [39]). Resistant accessions do not accumulate virus and present an OD equal or lower than the mean of the OD of the healthy *A. thaliana* Col-0 control. Susceptible accessions have an OD value that exceeds the healthy *A. thaliana* Col-0 control. The category to which each accession belongs is listed in Table 1 and Table S2, along with its OD values. Mock-inoculated plants were confirmed to be potyvirus-free.

### 4.6. Sample Processing

A biological replicate was composed by pooling up to four (2015) or three (2017) plants after a successful infection as measured by DAS ELISA. Aliquots of about 17 mg of fresh powder were weighted in 1.1-mL Micronic tubes and used in the targeted metabolite analysis. Samples were then lyophilized and aboveground dry mass determined with an analysis balance. Aliquots of about 10 mg were weighed in 1.1-mL Micronic tubes and used in the untargeted metabolite analysis.

### 4.7. Targeted Metabolite Analysis

Metabolites were extracted in a final volume of 650µL, twice with 80% (*v*/*v*) ethanol—HEPES/KOH 10mM (pH6) and once with 50% (*v*/*v*) ethanol—HEPES/KOH 10mM (ph6) [67]. Chlorophyll content was determined immediately after the extraction [68]. Glucose, fructose, sucrose [69] malate, fumarate ([70], glutamate [71] and amino acids [72] were determined in the supernatant. Starch [73] and protein [74] contents were determined on the pellet resuspended in 100 mM NaOH. Analyses were performed in 96-well microplates using Starlet pipetting robots (Hamilton), and absorbance was read in MP96 microplate readers (SAFAS). 

#### 4.7.1. Data Treatments

Metabolite data were normalized by dividing each measure by the value of a biological standard sample corresponding to a non-inoculated *A. thaliana* Col-0 sample harvested at the same time as the other samples. The final concentration of each metabolite is the average of the replicates of each accession. 

#### 4.7.2. Statistical Analysis

All principal component analysis (PCA), parametric and nonparametric statistical tests were performed using R version 3.4.0. Statistical significance was set at *p* < 0.05. Partial least squares regression (PLS) and orthogonal partial least-squares regression and discriminant analysis (OPLS-DA) were performed using the R packages mixOmics [75] and PLS [76].

### 4.8. Untargeted Metabolic Analysis

Untargeted metabolite measurements were conducted on the 26 accessions in the common garden experiment in 2015. Extraction was realized using a Starlet pipetting robot (Hamilton, Lancaster, PA, USA) with an extraction buffer composed of 80% (*v*/*v*) ethanol and 0.1% (*v*/*v*) formic acid, using methyl vanillate as an internal standard (50 µg/mL). All samples extracted were filtrated through a Multiscreen Solvinert 96-well filter plate (Merck Millipore, Burlington, MA, USA). 

#### 4.8.1. Quality Control (QC)

Twenty five µL of each sample were mixed together to generate a pooled quality control sample (QC). QC samples were analyzed every 10 injections to monitor and correct changes in the instrument response. Solvent blank samples (80% ethanol in water—0.1% formic acid) were also analyzed in-between the other samples. 

#### 4.8.2. Liquid Chromatography

Liquid chromatography was performed on a Dionex UHPLC Ultimate 3000 (Thermo Scientific, Waltham, MA, USA). Chromatographic separation was carried out in reverse-phase mode on a Gemini C18 column (2 × 150 mm; 3 µm, Phenomenex, Torrance, CA, USA) equipped with a Gemini C18 guard column (2 × 4 mm, Phenomenex). The mobile phase was composed of milliQ water with 0.1% formic acid (solvent A) and 100% Acetonitrile (solvent B) for a total run time of 18 min. The flow rate was 0.3 mL·min^−1^ and the column was heated to 30 °C. The autosampler temperature was maintained at 4 °C and the injection volume was 5 µL. 

#### 4.8.3. Mass Spectrometry

The UHPLC system was coupled with a LTQ-Orbitrap Elite mass spectrometer (Thermo Scientific). A heated electrospray interface was used and analyses were performed in positive mode. Acquisition was performed in full scan mode with a resolution of 240,000 FWHM in the scan range of *m*/*z* 50–1000. Data were recorded using the Xcalibur software (Thermo Scientific) and extracted with XCMS. 

#### 4.8.4. Data Processing and Statistical Analysis

Data were converted to mzXML file format. Peak picking and alignment were performed using XCMS in R [77]. XCMS-parameters were optimized as described by Patti and collaborators [78]. XCMS-data processing results in a data matrix which contains peak intensities that are a unique combination of retention-time and median *m*/*z* ratio. To exclude system-peaks (impurities in the measurement-system, visible in blanks) as well as poorly detected metabolic features, filter steps were performed based on QCs and blanks as described in TextS1. Statistical analysis was done in R version 3.4.0 and also using the web application BioStatFlow version 2.7.7 (http://biostatflow.org/ (accessed on 1 April 2021)) Thus, OPLS-DA (parameters: Kernel type linear, cross validation K-fold with K = 10, permutations for testing 100) were performed using the 505 features of the untargeted metabolic analysis combined with the 10 metabolites features assayed by targeted methods.

## Figures and Tables

**Table 1:** List of the *A. thaliana* accessions with their geographic position and their susceptible status to TuMV infection at 13 dai confirmed by OD values and its SD in the 2015 field experiment.

Genotype	ID ^1^	Latitude	Longitude	Country	OD Means	OD- Standard Deviations	Susceptibility Groups ^2^
Bay-0	6899	49	11	GER	0.004	0.0155	R
Mar2-3	159	47.35	3.93333	FRA	0.005	0.0087	R
MIB-39	190	47.3833	5.31667	FRA	0.007	0.0155	R
Petergof	7296	59	29	RUS	0.011	0.0252	R
Ra-0	6958	46	3.3	FRA	0.003	0.0072	R
Rak-2	8365	49	16	CZE	0.006	0.00204	R
TOU-A1-69	335	46.6667	4.11667	FRA	0.015	0.0224	R
TOU-A1-84	348	46.6667	4.11667	FRA	0.007	0.0204	R
Edi-0	6914	56	−3	UK	0.258	0.169	S
Bg-2	6709	47.6479	−122.305	USA	0.324	0.211	S
MIB-62	206	47.3833	5.31667	FRA	0.346	0.2084	S
Gu-0	6922	50.3	8	GER	0.389	0.272	S
Col-0	6909	38.3	−92.3	USA	0.391	0.215	S
Bu-0	8271	50.5	9.5	GER	0.4	0.203	S
TOU-A1-125	291	46.6667	4.11667	FRA	0.403	0.147	S
TOU-L-5	389	46.6667	4.11667	FRA	0.407	0.19	S
MIB-60	204	47.3833	5.31667	FRA	0.425	0.145	S
MIB-28	178	47.3833	5.31667	FRA	0.428	0.242	S
Hs-0	8310	52.24	9.44	GER	0.46	0.212	S
Mt-0	6939	32.34	22.46	LIB	0.467	0.2	S
MIB-20	171	47.3833	5.31667	FRA	0.525	0.294	S
MIB-67	210	47.3833	5.31667	FRA	0.549	0.249	S
TOU-A1-73	338	46.6667	4.11667	FRA	0.55	0.261	S
CUR-10	79	45	1.75	FRA	0.576	0.147	S
Mz-0	6940	50.3	8.3	GER	0.59	0.263	S
Bs-1	8270	47.5	7.5	SUI	0.66	0.339	S

^1^ ID according to [40]. ^2^ In 2015, categories have been defined according to the healthy control Col-0 which mean OD value was 0.088 (SD 0.01063). Infected genotypes with mean OD ≤ 0.088 were defined as resistant (R). Infected genotypes with mean OD > 2.5*0.088 were defined as susceptible [41].

**Table 2:** List of the variable importance in the projection (VIPs) identify by OPLS-DA analysis performed on TuMV-inoculated resistant and susceptible twenty-six accessions in 2015. For each VIP, comparisons between resistant (R) and susceptible (S) accessions were done. The fold change was calculated for each VIP. Primary metabolites are light-grey highlighted. Metabolites that accumulate significantly more in resistant accessions are at the bottom of the table.

VIP OPLS-DA ^1^	VIP Values	*m*/*z*^2^	rt ^3^	Resistant vs. Susceptible Metabolic Contents	Fold Change
Sucrose	2.213	NA	NA	S > R *** ^4^	2.89
303;463	1.973	303.133	463.27	S > R ***	3.69
356;452	1.958	356.12	452.352	S > R ***	2.17
219;311	1.94	219.101	311.134	S > R ***	4.81
533;392	1.926	533.155	391.765	S > R ***	4.98
332;430	1.898	332.132	430.481	S > R ***	3.78
116;100	1.867	116.07	100.163	S > R ***	6.05
205;242	1.837	205.097	242.121	S > R ***	2.51
103;242	1.799	103.041	242.089	S > R ***	2.86
302;506	1.796	302.102	506.314	S > R ***	5.76
255;544	1.769	255.112	543.799	S > R ***	9.96
221;216	1.748	221.092	215.744	S > R ***	3.13
385;210	1.738	385.106	210.083	S > R ***	45.77
Glucose	1.734	NA	NA	S > R ***	2.49
175;402	1.713	175.148	401.831	S > R ***	3.77
903;319	1.71	903.277	318.889	S > R ***	8.48
503;391	1.695	503.19	391.457	S > R ***	2.51
343;345	1.693	343.117	345.319	S > R ***	2.08
Fructose	1.683	NA	NA	S > R ***	2.49
474;107	1.675	474.218	107.298	S > R ***	2.68
315;448	1.674	315.133	447.821	S > R ***	2.39
209;488	1.669	209.153	488.35	S > R ***	2.7
209;414	1.663	209.153	413.756	S > R ***	2.14
212;284	1.657	211.559	283.891	S > R ***	1.56
543;99	1.645	543.132	98.5221	S > R ***	5.84
124;346	1.638	124.075	346.354	S > R ***	1.92
370;302	1.61	370.149	301.519	S > R ***	28.49
203;204	1.591	203.084	203.918	S > R ***	8.24
Glutamate	1.59	NA	NA	S > R ***	1.47
430;333	1.581	430.17	332.878	S > R ***	3.3
331;329	1.578	331.117	328.817	S > R ***	1.58
226;406	1.554	226.107	406.369	S > R ***	7.97
151;359	1.549	151.075	359.377	S > R ***	1.78
449;319	1.545	449.106	318.949	S > R ***	1.75
757;319	1.537	757.217	319.13	S > R ***	1.69
270;587	1.534	270.133	587.091	S > R ***	43.68
315;370	1.524	315.133	370.476	S > R ***	3.93
162;402	1.511	162.055	402.281	S > R ***	1.9
221;230	1.503	221.121	230.244	S > R ***	1.74
394;517	1.49	394.204	517.072	S > R ***	17.28
302;407	1.486	302.101	406.697	S > R ***	7.16
482;101	1.475	482.107	101.274	S > R ***	1.79
Amino Acids	1.462	NA	NA	S > R ***	1.88
355;306	1.451	355.102	306.288	S > R ***	3.29
191;416	1.448	191.143	416.467	S > R ***	2.49
193;324	1.445	193.125	323.817	S > R ***	1.46
149;360	1.437	149.096	360.18	S > R ***	1.66
642;445	1.435	642.254	445.078	S > R ***	2
321;760	1.419	321.114	760.442	S > R ***	3.7
182;466	1.415	182.081	465.796	S > R ***	4.21
305;210	1.408	305.086	210.166	S > R ***	2.24
348;319	1.396	348.274	318.945	S > R ***	3.61
164;496	1.375	164.07	496.307	S > R ***	2.22
165;424	1.372	165.127	424.14	S > R ***	1.54
317;464	1.37	317.101	463.65	S > R ***	1.25
191;363	1.36	191.07	362.643	S > R ***	2.76
219;486	1.34	219.101	486.477	S > R ***	2.7
105;700	1.33	105.069	700.287	S > R ***	1.59
367;358	1.323	367.101	358.491	S > R ***	1.69
201;344	1.319	201.054	343.832	S > R ***	1.71
189;325	1.312	189.127	324.754	S > R ***	1.74
146;242	1.311	146.06	241.843	S > R ***	2.31
133;296	1.307	133.064	295.68	S > R ***	2.65
179;604	1.306	179.106	603.961	S > R ***	1.64
374;322	1.305	374.144	321.703	S > R ***	1.77
373;453	1.29	373.127	452.6	S > R ***	1.81
302;382	1.282	302.041	381.707	S > R ***	1.54
161;442	1.274	161.096	441.797	S > R ***	1.79
164;375	1.269	164.07	375.012	S > R ***	2.95
109;359	1.267	109.064	359.34	S > R ***	1.92
386;278	1.256	386.22	277.549	S > R ***	3.48
391;774	1.255	391.245	774.477	S > R ***	2.29
409;491	1.251	409.169	490.545	S > R ***	27.12
291;259	1.241	291.181	259.063	S > R ***	4.78
192;462	1.227	192.041	462.37	S > R ***	1.27
107;370	1.219	107.085	370.001	S > R ***	1.39
420;276	1.217	419.695	275.797	S > R ***	1.77
193;373	1.189	193.086	373.266	S > R ***	2.04
379;402	1.179	379.095	402.311	S > R ***	2.88
181;464	1.176	181.086	463.916	S > R ***	1.42
181;328	1.17	181.086	327.894	S > R ***	1.68
80;395	1.168	80.049	395.289	S > R ***	1.57
373;344	1.16	373.127	343.531	S > R ***	1.54
627;299	1.156	627.155	299.369	S > R ***	2.8
96;389	1.155	96.08	389.004	S > R ***	1.74
195;453	1.154	195.065	452.614	S > R ***	1.55
79;396	1.152	79.041	395.754	S > R ***	1.52
105;327	1.15	105.069	326.898	S > R ***	1.72
210;465	1.147	210.112	464.799	S > R ***	2.44
335;506	1.143	335.127	506.441	S > R ***	3.71
86;126	1.114	86.096	126.246	S > R ***	2.09
396;257	1.106	396.185	256.51	S > R ***	1.43
611;354	1.102	611.158	353.603	S > R ***	1.34
178;191	1.102	178.089	191.013	S > R ***	3.43
169;496	1.101	169.049	495.5	S > R ***	3.67
162;249	1.098	162.055	249.101	S > R ***	1.5
396;348	1.092	396.115	347.739	S > R ***	4.14
103;389	1.086	103.054	389.444	S > R ***	2
521;325	1.084	521.201	325.328	S > R ***	2.52
133;126	1.072	133.105	126.347	S > R **	2.32
209;426	1.071	209.153	425.939	S > R ***	1.98
162;357	1.069	162.055	356.879	S > R ***	1.74
201;451	1.063	201.054	451.123	S > R ***	1.75
527;460	1.056	527.103	460.095	S > R ***	2.02
Fumarate	1.054	NA	NA	S > R ***	1.38
393;416	1.052	393.188	415.816	S > R ***	1.71
212;774	1.052	212.094	774.047	S > R ***	2.76
402;420	1.048	402.162	419.597	S > R **	1.87
404;388	1.038	404.227	388.155	S > R **	1.31
464;367	1.035	464.248	366.989	S > R ***	1.97
225;371	1.034	225.148	370.798	S > R ***	1.57
103;284	1.033	103.054	283.823	S > R ***	3.26
227;789	1.023	227.163	788.506	S > R ***	1.61
222;216	1.016	221.602	215.822	S > R **	1.33
367;342	1.011	367.153	341.908	S > R ***	1.96
302;463	1.006	302.102	463.078	S > R ***	1.49
309;325	1.003	309.116	324.659	S > R **	1.44
244;325	1.001	244.096	324.677	S > R **	1.41
351;184	2.047	351.006	184.222	R > S ***	2.88
432;184	1.831	431.971	183.776	R > S ***	3.44
137;131	1.618	136.931	131.31	R > S ***	1.39
512;491	1.563	512.127	491.367	R > S ***	2.54
394;108	1.548	394.2	108.419	R > S ***	1.93
337;573	1.51	337.292	572.917	R > S ***	1.64
299;181	1.396	299.098	181.363	R > S ***	22.3
181;139	1.293	181.053	138.934	R > S ***	1.94
324;184	1.263	323.989	183.797	R > S ***	106.8
280;184	1.229	280.084	183.768	R > S ***	5.9
361;491	1.218	361.092	490.564	R > S ***	2.07
155;132	1.141	154.941	131.541	R > S ***	1.33
433;482	1.114	433.112	481.588	R > S ***	244
433;700	1.109	433.241	700.261	R > S ***	1.82
64;395	1.091	63.934	395.047	R > S ***	3.6
256;668	1.089	256.08	667.874	R > S ***	2.12
460;740	1.076	460.269	740.005	R > S **	1.42
449;442	1.069	449.107	442.311	R > S ***	6.17
244;130	1.049	243.942	130.167	R > S ***	1.35
347;240	1.039	347.159	239.659	R > S **	1.48
86;184	1.015	86.059	184.278	R > S ***	1.87

^1^ When undetermined, VIP are identified through mz/rt values. VIP values are classified in decreasing order, ^2^ mass to charge ratio, ^3^ retention time. ^4^ The significance was assessed through a Wilcoxon test at *** *p* < 0.001, ** 0.001 < *p* < 0.01.

## Data Availability

Publicly available datasets were analyzed in this study. This data can be found here: https://data.inrae.fr/privateurl.xhtml?token=7ddc25dc-e76c-4ac4-9acc-61d89e64076f (accessed on 1 April 2021).

## Supplementary Materials

The following are available online at https://www.mdpi.com/article/10.3390/metabo11040230/s1, Figure S1: Principal component analysis performed on the 505 metabolic signatures (*m*/*z*) measured on 26 *A. thaliana* accessions in the ‘2015’ field experiment, Figure S2: OPLS-DA analysis and its parameters of validation for the TuMV-inoculated resistant and susceptible 26 *A. thaliana* accessions of the ‘2015’ field experiment, Figure S3: Climate raw data and comparison between the 2015 and 2017 field experiment, Table S1: Spearman correlations between dry biomass (DB), viral accumulation (OD) and 10 primary metabolites content measured on the 26 *A. thaliana* accessions of the ‘2015’ field experiment , Table S2: List of the *A. thaliana* accessions with their geographic position and their susceptible status to TuMV infection confirmed by OD values and SD in the ‘2017’ field experiment, Table S3: Common Variable Importance in the Projection (VIPs) obtained with the OPLS-DA performed on mock and TuMV-inoculated and on resistant and susceptible accessions of twenty-six accessions in 2015, Table S4: Comparisons between the Variable Importance in the Projection (VIPs) identify by OPLS-DA analysis and those identify by PLS analysis performed with resistant and susceptible twenty-six accessions in 2015.
