# Supplementary material for: Metabolic Profile Discriminates and Predicts Arabidopsis Susceptibility to Virus under Field Conditions
Source: Metabolites. 2021 Apr 9;11(4):230. doi: 10.3390/metabo11040230 (PMC8069729; doi:10.3390/metabo11040230)
Supplement: Supplementary file 1 [file metabolites-11-00230-s001.pdf]

Supplementary Materials

Article title: **Metabolic profile discriminates and predicts *Arabidopsis* susceptibility to virus under field conditions**

Table S1 Spearman correlations between dry biomass (DB), viral accumulation (OD) and 10 primary metabolites content measured on the 26 *A. thaliana* accessions of the '2015' field experiment

	OD	Amino Acids	Proteins	Glutamate	Malate	Fumarate	Starch	Glucose	Fructose	Sucrose	Chla
DB_Mock-inoculated	-	-0.66 ***	-0.42 *	-0.45 *	0.38 *	0.36 .	0.48 **	0.65 ***	0.18	0.3	-0.12 NS
DB_TuMV-inoculated	-0.62 ***	- 0.75 ***	-0.27 NS	-0.71 ***	-0.44 *	-0.22 NS	-0.3 NS	-0.38 *	-0.61 ***	-0.52 ***	0.23 NS
OD_TuMV-inoculated	1	0.49 ***	0.35 .	0.63 ***	0.22	0.44 *	0.58 **	0.55 ***	0.42 *	0.70 ***	-0.08 NS

All the significant correlations where showed by asterisks and values (Signif.codes: 0 '***' 0.001 '**' 0.01 '*' 0.05 '.' 0.1)
- Non tested and NS = Non significant differences

Table S2 List of the A. thaliana accessions with their geographic position and their susceptible status to TuMV infection confirmed by OD values and SD in '2017' experiment

Genotype	ID[1]	Latitude	Longitude	Country	Suceptibility Groups[2]	OD Means	OD-Standard Deviations
Ra-0	6958	46	3.3	FRA	R	0.0188	0.0174
TOU-A1-84	348	46.6667	4.11667	FRA	R	0.0202	0.02
Bay-0	6899	49	11	GER	R	0.0218	0.0143
Petergof	7296	59	29	RUS	R	0.0406	0.0197
Rak-2	8365	49	16	CZE	R	0.053	0.0037
Col-0	6909	38.3	-92.3	USA	S	0.325	0.165
Mt-0	6939	32.34	22.46	LIB	S	0.379	0.122
Bu-0	8271	50.5	9.5	GER	S	0.621	0.222
CUR-10	79	45	1.75	FRA	S	0.769	0.274
Bs-1	8270	47.5	7.5	SUI	S	0.832	0.364

[1] ID according to [40].
[2] In 2017, categories have been defined according to the healthy control Col-0 which mean OD value was 0.078 (SD 0.00722). Infected genotypes with mean OD ≤ 0.078 were defined as resistant (R). Infected genotypes with mean OD>2.5*0.078 were defined as susceptible [41].

Table S3 Common Variable Importance in the Projection (VIPs) obtained with the OPLS-DA performed on mock and TuMV-inoculated and on resistant and susceptible accessions of twenty-six accessions in 2015. For each VIP, the comparison between the metabolic content in mock (M) and TuMV-inoculated (I) samples was done and the fold change was represented. Moreover, the comparison, for each VIP, of metabolic content between resistant (R) and susceptible (S) accessions was also represented.

VIP-OPLS-DA[1]	VIP values	m/z[2]	rt[3]	Mock vs. TuMV Infected	Fold change	Resistant vs. Susceptible
**193;324**	**2.201000988**	193.13	323.817	I > M	**4.54**	S > R ***[4]
**331;329**	**2.177409793**	331.12	328.817	I > M	**2.55**	S > R ***
**474;107**	**2.100348101**	474.22	107.298	I > M	**7.12**	S > R ***
**482;101**	**2.02851797**	482.11	101.274	I > M	**3.24**	S > R ***
**205;242**	**1.95494017**	205.1	242.121	I > M	**3.16**	S > R ***
**209;488**	**1.895244441**	209.15	488.35	I > M	**3.87**	S > R ***
**212;284**	**1.812272811**	211.56	283.891	I > M	**1.97**	S > R ***
**343;345**	**1.793276838**	343.12	345.319	I > M	**2.41**	S > R ***
**116;100**	**1.77949346**	116.07	100.163	I > M	**6.88**	S > R ***
**162;357**	**1.767230127**	162.05	356.879	I > M	**3.1**	S > R ***
**303;463**	**1.682969043**	303.13	463.27	I > M	**2.9**	S > R ***
**219;311**	**1.640982077**	219.1	311.134	I > M	**3.44**	S > R ***
**355;306**	**1.594996482**	355.1	306.288	I > M	**8.11**	S > R ***
**305;210**	**1.575910966**	305.09	210.166	I > M	**2.69**	S > R ***
**133;296**	**1.573543164**	133.06	295.68	I > M	**3.79**	S > R ***
**221;216**	**1.49354017**	221.09	215.744	I > M	**2.46**	S > R ***
**124;346**	**1.493002053**	124.08	346.354	I > M	**1.68**	S > R ***
**503;391**	**1.478656897**	503.19	391.457	I > M	**2.24**	S > R ***
**373;344**	**1.456529478**	373.13	343.531	I > M	**1.78**	S > R ***
**151;359**	**1.45485619**	151.08	359.377	I > M	**1.28**	S > R ***
**374;322**	**1.444521384**	374.14	321.703	I > M	**1.88**	S > R ***
**162;249**	**1.431791986**	162.05	249.101	I > M	**1.81**	S > R ***
**175;402**	**1.423091039**	175.15	401.831	I > M	**2.52**	S > R ***
**332;430**	**1.416086097**	332.13	430.481	I > M	**2.32**	S > R ***
**315;448**	**1.412434025**	315.13	447.821	I > M	**2.05**	S > R ***
**221;230**	**1.4121623**	221.12	230.244	I > M	**1.67**	S > R ***
**391;774**	**1.397491479**	391.24	774.477	I > M	**2.36**	S > R ***
**386;278**	**1.385282143**	386.22	277.549	I > M	**5.51**	S > R ***
**348;319**	**1.376257143**	348.27	318.945	I > M	**4.2**	S > R ***
**356;452**	**1.358448769**	356.12	452.352	I > M	**1.69**	S > R ***
**201;344**	**1.355039751**	201.05	343.832	I > M	**1.71**	S > R ***

**222;216**	**1.351138929**	221.6	215.822	**I > M**	**2.46**	**S > R** **
**370;302**	**1.331366534**	370.15	301.519	**I > M**	**5.59**	**S > R** ***
**315;370**	**1.330904866**	315.13	370.476	**I > M**	**3.58**	**S > R** ***
**367;358**	**1.251165644**	367.1	358.491	**I > M**	**1.62**	**S > R** ***
**182;466**	**1.243641489**	182.08	465.796	**I > M**	**3.65**	**S > R** ***
**302;407**	**1.221943812**	302.1	406.697	**I > M**	**4.3**	**S > R** ***
**191;363**	**1.219391802**	191.07	362.643	**I > M**	**2.37**	**S > R** ***
**367;342**	**1.199530791**	367.15	341.908	**I > M**	**2.4**	**S > R** ***
**291;259**	**1.165429681**	291.18	259.063	**I > M**	**3.68**	**S > R** ***
**178;191**	**1.128214863**	178.09	191.013	**I > M**	**3.11**	**S > R** ***
**396;348**	**1.126209569**	396.11	347.739	**I > M**	**7.35**	**S > R** ***
**394;517**	**1.125664243**	394.2	517.072	**I > M**	**4.36**	**S > R** ***
**543;99**	**1.122326073**	543.13	98.5221	**I > M**	**2.62**	**S > R** ***
**212;774**	**1.113329112**	212.09	774.047	**I > M**	**3.14**	**S > R** ***
**164;375**	**1.107007943**	164.07	375.012	**I > M**	**2.38**	**S > R** ***
**210;465**	**1.09694612**	210.11	464.799	**I > M**	**2.27**	**S > R** ***
**379;402**	**1.091061829**	379.09	402.311	**I > M**	**2.47**	**S > R** ***
**133;126**	**1.06741008**	133.1	126.347	**I > M**	**2.62**	**S > R** **
**409;491**	**1.066982099**	409.17	490.545	**I > M**	**1.46**	**S > R** ***
**109;359**	**1.035812334**	109.06	359.34	**I > M**	**1.67**	**S > R** ***
**162;402**	**1.032043124**	162.05	402.281	**I > M**	**1.41**	**S > R** ***
**227;789**	**1.023548533**	227.16	788.506	**I > M**	**1.71**	**S > R** ***
**192;462**	**1.000060126**	192.04	462.37	**I > M**	**1.31**	**S > R** ***
**433;700**	**2.253892757**	433.24	700.261	**I > M**	**3**	**R > S** ***
**361;491**	**1.914433317**	361.09	490.564	**I > M**	**4.84**	**R > S** ***
**512;491**	**1.813252344**	512.13	491.367	**I > M**	**21.08**	**R > S** ***
**64;395**	**1.668700459**	63.934	395.047	**I > M**	**1.25**	**R > S** ***
**79;396**	**2.194133112**	79.041	395.754	**M > I**	**1.85**	**S > R** ***
**105;700**	**1.816365199**	105.07	700.287	**M > I**	**1.88**	**S > R** ***
**169;496**	**1.288454553**	169.05	495.5	**M > I**	**1.91**	**S > R** ***
**449;442**	**1.102028381**	449.11	442.311	**M > I**	**4.26**	**R > S** ***
**137;131**	**1.036410357**	136.93	131.31	**M > I**	**1.35**	**R > S** ***

[1] When undetermined, VIP are identified through m/z;rt values. VIP values are classified in decreasing order.
[2] mass to charge ratio
[3] retention time
[4] The significance was assessed through a Wilcoxon test at *** $P < 0.001$, ** $0.001 < P < 0.01$

Table S4 Comparisons between the Variable Importance in the Projection (VIPs) identify by OPLS-DA analysis and those identify by PLS analysis performed with resistant and susceptible twenty-six accessions in 2015. The same results of metabolic content comparisons were found between resistant (R) and susceptible (S) accessions. The fold change was also represented for each VIP. Primary metabolites are light-grey highlighted. Metabolites that accumulate significantly more in resistant accessions are at the bottom of the table.

VIP PLS[1]	VIP-PLS value	VIP-OPLS-DA value	m/z[2]	rt[3]	Resistant vs Susceptible metabolic contents	Fold change
356;452	2.1040	1.9580	356.1202	452.352	S > R ***[4]	2.17
Glutamate	2.0854	1.5902	NA	NA	S > R ***	1.47
Sucrose	1.9944	2.2126	NA	NA	S > R ***	2.89
219;311	1.9753	1.9395	219.1011	311.134	S > R ***	4.81
385;210	1.9524	1.7380	385.1055	210.083	S > R ***	45.77
373;453	1.9059	1.2900	373.1273	452.6	S > R ***	1.81
270;587	1.8532	1.5343	270.133	587.091	S > R ***	43.68
315;370	1.8277	1.5236	315.1333	370.476	S > R ***	3.93
348;319	1.8190	1.3963	348.2736	318.945	S > R ***	3.61
394;517	1.8096	1.4897	394.2045	517.072	S > R ***	17.28
191;363	1.7958	1.3599	191.0699	362.643	S > R ***	2.76
474;107	1.7890	1.6748	474.2178	107.298	S > R ***	2.68
226;406	1.7712	1.5540	226.1066	406.369	S > R ***	7.97
420;276	1.7295	1.2167	419.6949	275.797	S > R ***	1.77
367;342	1.7210	1.0107	367.1531	341.908	S > R ***	1.96
302;407	1.6916	1.4864	302.1013	406.697	S > R ***	7.16

503;391	1.6879	1.6947	503.1902	391.457	S > R ***	2.51
116;100	1.6790	1.8669	116.0701	100.163	S > R ***	6.05
396;257	1.6725	1.1064	396.1854	256.51	S > R ***	1.43
303;463	1.6577	1.9734	303.1334	463.27	S > R ***	3.69
355;306	1.6414	1.4506	355.1015	306.288	S > R ***	3.29
370;302	1.6342	1.6097	370.1486	301.519	S > R ***	28.49
343;345	1.6096	1.6930	343.1168	345.319	S > R ***	2.08
533;392	1.6000	1.9258	533.1549	391.765	S > R ***	4.98
201;451	1.5993	1.0635	201.0543	451.123	S > R ***	1.75
757;319	1.5938	1.5374	757.2171	319.13	S > R ***	1.69
386;278	1.5899	1.2560	386.2198	277.549	S > R ***	3.48
903;319	1.5874	1.7102	903.2769	318.889	S > R ***	8.48
449;319	1.5823	1.5451	449.1063	318.949	S > R ***	1.75
182;466	1.5645	1.4146	182.0808	465.796	S > R ***	4.21
175;402	1.5414	1.7128	175.1478	401.831	S > R ***	3.77
305;210	1.5354	1.4081	305.0861	210.166	S > R ***	2.24
195;453	1.5352	1.1541	195.0648	452.614	S > R ***	1.55
221;216	1.5350	1.7483	221.0915	215.744	S > R ***	3.13
374;322	1.5256	1.3048	374.1436	321.703	S > R ***	1.77
Glucose	1.5212	1.7342	NA	NA	S > R ***	2.49
179;604	1.5211	1.3057	179.1063	603.961	S > R ***	1.64

161;442	1.5180	1.2744	161.0957	441.797	S > R ***	1.79
**394;108**	1.7935	**1.5477**	**394.2001**	**108.419**	**R > S *** **	**1.93**
**351;184**	1.7480	**2.0467**	**351.0062**	**184.222**	**R > S *** **	**2.88**
**324;184**	1.7384	**1.2634**	**323.9889**	**183.797**	**R > S *** **	**106.8**
**280;184**	1.7056	**1.2289**	**280.0842**	**183.768**	**R > S *** **	**5.9**
**432;184**	1.6488	**1.8309**	**431.9707**	**183.776**	**R > S *** **	**3.44**
**86;184**	1.6112	**1.0146**	**86.05939**	**184.278**	**R > S *** **	**1.87**

[1] When undetermined, VIP are identified through m/z;rt values. VIP values are classified in decreasing order.
[2] mass to charge ratio
[3] retention time
[4] The significance was assessed through a Wilcoxon test at *** *P* < 0.001, ** 0.001 < P < 0.01

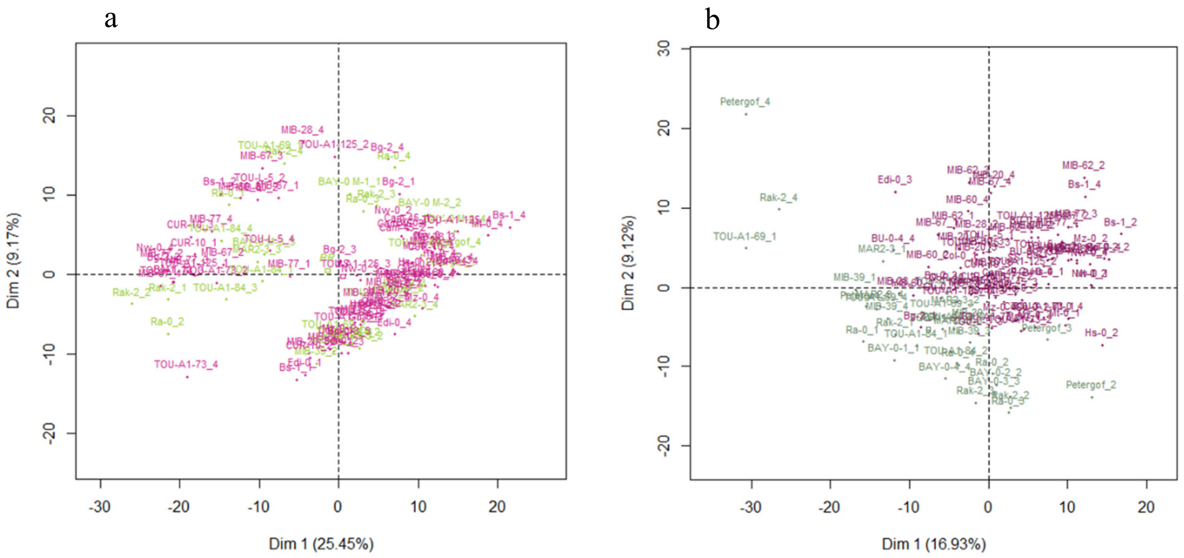

Figure S1. Principal component analysis performed on the 505 metabolic signatures (m/z) measured on 26 *A. thaliana* accessions in the '2015' field experiment

a. PCA performed on the 505 metabolic signatures (m/z) measured by UHPLC-LTQ Orbitrap on 26 *A. thaliana* mock-inoculated accessions. The two major components that together accounted for 34.62% of the variance. Resistant and susceptible accessions are in light green and light pink, respectively. b. PCA performed on the 505 metabolic signatures (m/z) measured by UHPLC-LTQ Orbitrap on 26 *A. thaliana* TUMV-inoculated accessions. The two major components accounted for 26.05% of the variance. Resistant and susceptible accessions are in dark green and dark pink,

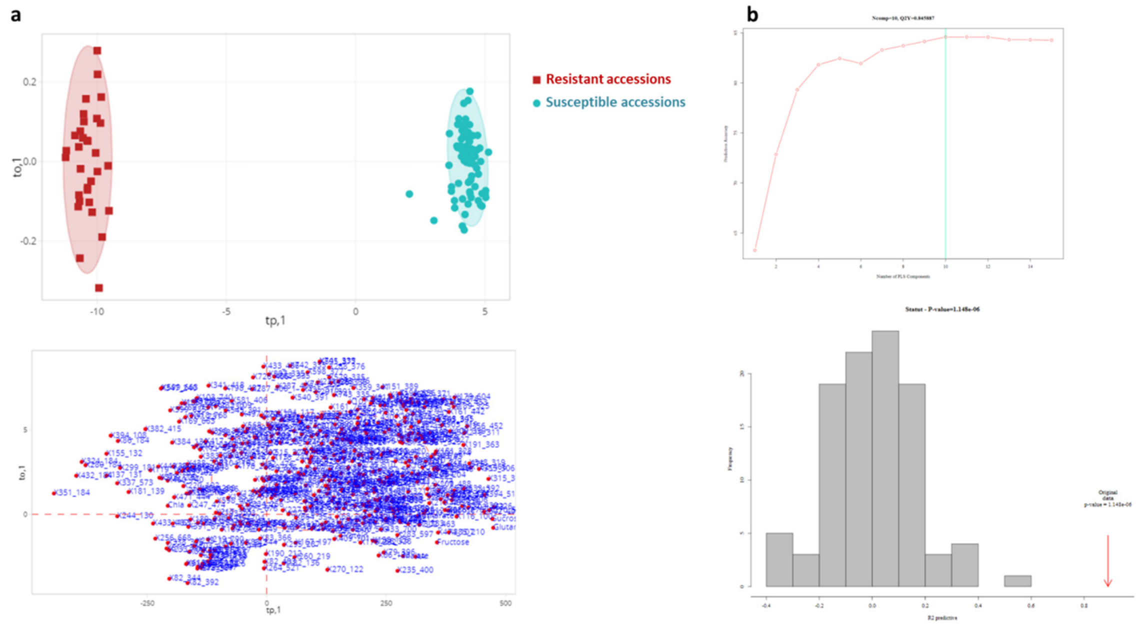

Figure S2 OPLS-DA analysis and its parameters of validation for the TuMV-inoculated resistant and susceptible 26 *A. thaliana* accessions of the '2015' field experiment a. OPLS-DA results with the score plot and the loading plot containing all the metabolic variables tested. The resistant (in red) and susceptible (in blue) accessions were represented. b. Parameters for validation of the OPLS-DA analysis.

a

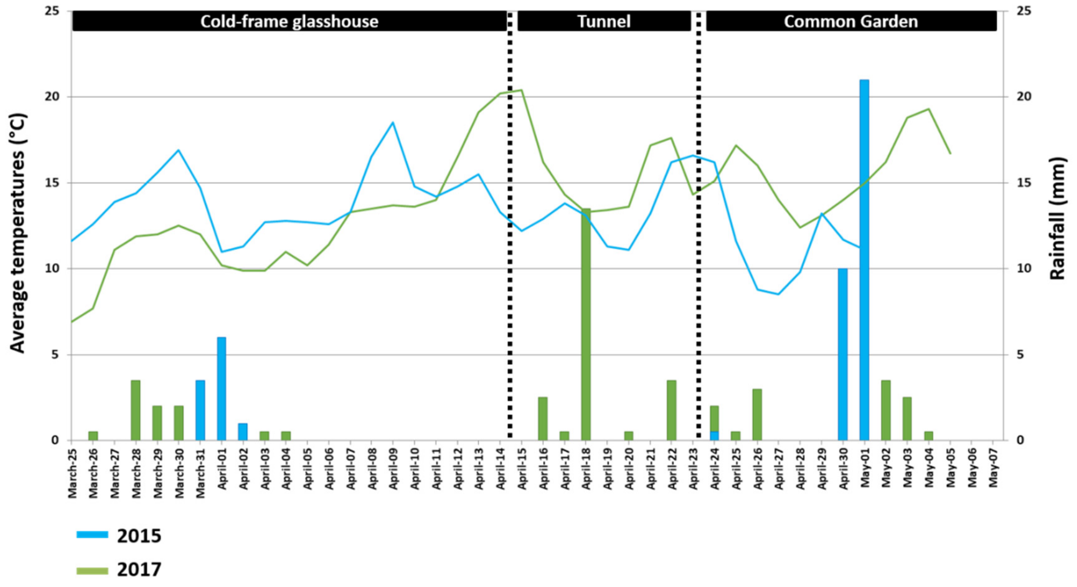
Cold-frame glasshouse
Tunnel
Common Garden
Average temperatures (°C)
Rainfall (mm)
2015
2017

b

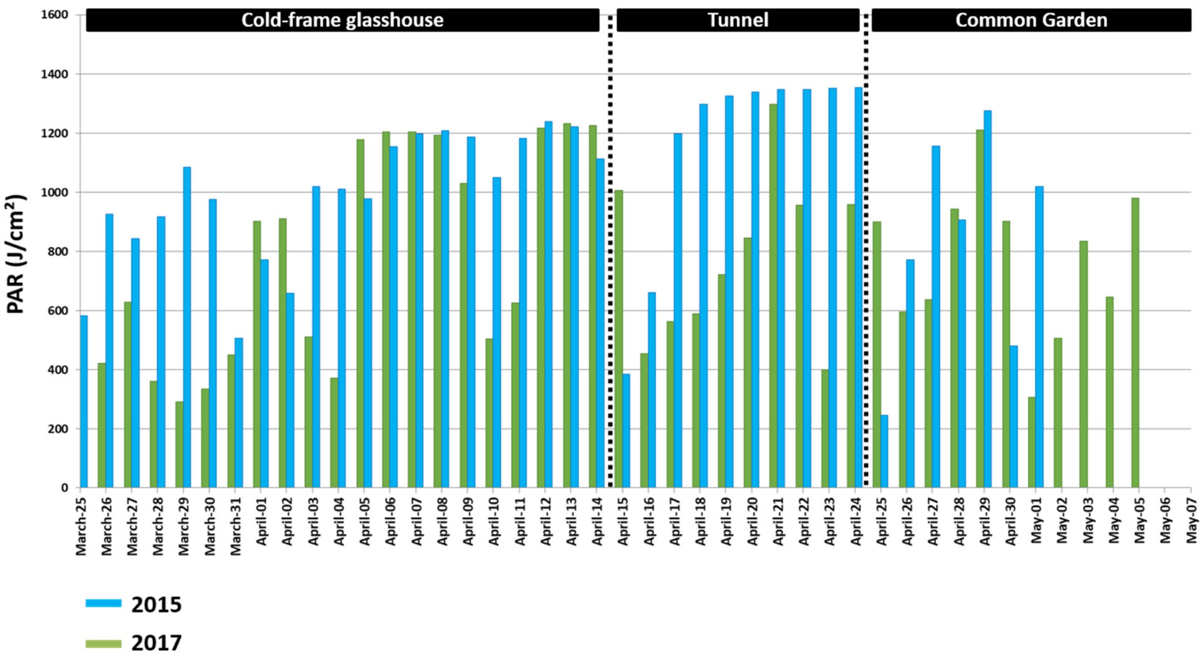
Cold-frame glasshouse
Tunnel
Common Garden
PAR (J/cm²)
2015
2017

c

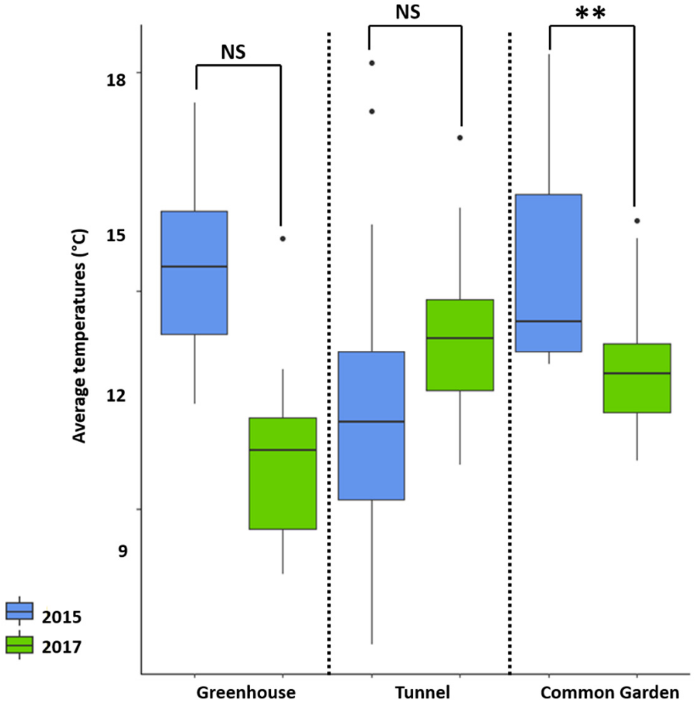

d

e

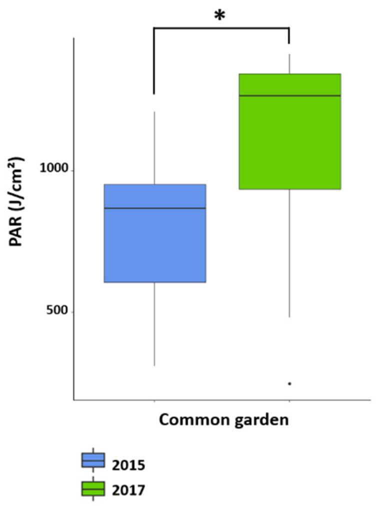

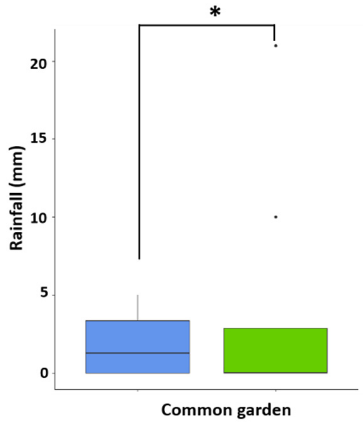

Figure S3 Climate raw data and comparison between the 2015 and 2017 field experiment a. Rainfall and average temperature in 2015 and 2017 b. PAR in 2015 and 2017 recorded under the three successive conditions of the experiment, cold-frame glasshouse, tunnel and

common garden. c. Comparison of the average temperatures between 2015 and 2017 under the 3 successive conditions of the experiments. d. Comparison of PAR between 2015 and 2017 under common garden conditions. e. Comparison of rainfall between 2015 and 2017 under common garden conditions. The significance was assessed through a Wilcoxon test at *p-value* = 0.05 indicated by a *, NS: non-significant.
